# Effects of Baicalin on piglet monocytes involving PKC–MAPK signaling pathways induced by *Haemophilus parasuis*

**DOI:** 10.1186/s12917-019-1840-x

**Published:** 2019-03-25

**Authors:** Chun Ye, Ruizhi Li, Lei Xu, Yinsheng Qiu, Shulin Fu, Yu Liu, Zhongyuan Wu, Yongqing Hou, Chien-An Andy Hu

**Affiliations:** 1 Hubei Key Laboratory of Animal Nutrition and Feed Science, Wuhan Polytechnic University, Wuhan, 430023 People’s Republic of China; 2 Hubei Collaborative Innovation Center for Animal Nutrition and Feed Safety, Wuhan, 430023 People’s Republic of China; 3 Biochemistry and Molecular Biology, University of New Mexico School of Medicine, Albuquerque, NM 87131 USA

**Keywords:** Baicalin, *Haemophilus parasuis*, PKC–MAPK, Apoptosis, Glässer’s disease

## Abstract

**Background:**

*Haemophilus parasuis* (HPS) is the causative agent of Glässer’s disease, characterized by arthritis, fibrinous polyserositis and meningitis, and resulting in worldwide economic losses in the swine industry. Baicalin (BA), a commonly used traditional Chinese medication, has been shown to possess a series of activities, such as anti-bacterial, anti-viral, anti-tumor, anti-oxidant and anti-inflammatory activities. However, whether BA has anti-apoptotic effects following HPS infection is unclear. Here, we investigated the anti-apoptotic effects and mechanisms of BA in HPS-induced apoptosis via the protein kinase C (PKC)–mitogen-activated protein kinase (MAPK) pathway in piglet’s mononuclear phagocytes (PMNP).

**Results:**

Our data demonstrated that HPS could induce reactive oxygen species (ROS) production, arrest the cell cycle and promote apoptosis via the PKC–MAPK signaling pathway in PMNP. Moreover, when BA was administered, we observed a reduction in ROS production, suppression of cleavage of caspase-3 in inducing apoptosis, and inhibition of activation of the PKC–MAPK signaling pathway for down-regulating p-JNK, p-p38, p-ERK, p-PKC-α and PKC-δ in PMNP triggered by HPS.

**Conclusions:**

Our data strongly suggest that BA can reverse the apoptosis initiated by HPS through regulating the PKC–MAPK signaling pathway, which represents a promising therapeutic agent in the treatment of HPS infection.

## Background

*Haemophilus parasuis* (HPS) is the causative agent of Glässer’s disease, which is responsible for cases of arthritis, fibrinous polyserositis and meningitis affecting piglets worldwide and could cause acute septicemia in non-immune high-health status pigs of all ages [1]. HPS has become one of the most important bacterial pathogens, resulting in major economic losses to the swine industry [2]. The knowledge about the mechanism by which the bacterium interacts with the host and causes pathogenicity is very sparse and remains controversial [1]. Cytolethal distending toxin of HPS can lead to distension, G2 arrest and apoptosis of host cells in kidney epithelial (PK-15) and porcine alveolar macrophage (PAM) cells [2]. HPS is able to up-regulate several genes related to inflammation and phagocytosis, and several pro-inflammatory cytokines in PAM cells [3]. Our previous studies demonstrated that HPS infection could induce production of reactive oxygen species (ROS) and promote apoptosis of piglet’s mononuclear phagocytes (PMNP) via the nuclear factor-kappa B (NF-κB) signaling pathway [4].

Vaccines against HPS have been used to prevent infection, but they are limited to either serovar or strain of bacteria, and an effective vaccine providing cross-protection against all serovars is desirable [5]. Antibiotics are commonly used to treat the disease in the industry [6], but long-term inappropriate use increases antibiotic resistance and residues, leading to difficulties in preventing and controlling clinical infections in the future [7]. Therefore, it has become an increasingly important issue in drug development to find anti-bacterial, anti-inflammatory and immune-regulatory drugs with definite curative effects against HPS as well as less adverse reactions.

Many natural compounds, such as flavonoids, have been investigated as important natural resources to overcome this problem [8]. Baicalin (BA) is a flavonoid glycoside isolated from the roots of *Scutellaria baicalensis* Georgi. A number of studies have demonstrated anti-bacterial, anti-viral, anti-tumor, anti-oxidant and anti-inflammatory properties of BA in both animals and humans [9, 10]. Earlier studies by our group showed that BA can suppress the NLRP3 inflammasome pathway under LPS stimulation [11], and it also has anti-inflammatory activity via NF-κB and NLRP3 inflammasome signaling evoked by HPS in PMNP [4]. Furthermore, HPS can activate NF-κB and mitogen-activated protein kinase (MAPK) pathways mediated by toll-like receptors (TLRs) in host cells [12].

It has been reported that protein kinase C (PKC) can activate MAPK signaling in various cells under different stimulation. ROS can activate the MAPK pathway via PKC mediation, while PKC acts as a key signaling node in the aldose reductase inhibitor-mediated anti-inflammatory mechanism, and might act as a crucial link between ROS production and subsequent NF-κB and MAPK signaling activations [13]. Activator protein-1 (AP-1) mediates gene expression, transformation, apoptosis, pulmonary defense, inflammation and immune responses [14] and is predominantly regulated by MAPK family members such as JNK, p38 and ERK [15–17]. However, activated PKCs may also participate in transcriptional regulation of AP-1. The regulation of PKC significantly reduces asbestos-induced c-Fos or c-Jun mRNA expression in rat pleural mesothelial cells and R6 rat fibroblasts, separately [18, 19]. PKCs could participate in LPS-induced c-Fos and c-Jun expression in human smooth muscle cells by phosphorylation of MAPKs. In addition, p42/p44 MAPK was shown to be activated by PKC in response to LPS in human smooth muscle cells [14]. However, after HPS stimulated the regulation of AP-1 through PKCs-dependent MAPKs activation was also unclear in PMNP.

As mentioned above, more information is required to better understand the responses of PMNP to HPS infection. Understanding the cellular signaling pathways leading to apoptosis in response to HPS infection may provide new therapeutic strategies for treatment. Considering the above facts, the present study aimed to examine ROS generation, cell-cycle arrest and cell apoptosis induction by HPS infection and the protective effect of BA on these. Importantly, we aimed to demonstrate that HPS-induced apoptosis was regulated by an increase in ROS production and the activation of PKC–MAPK pathways – this should simultaneously show inhibition by BA on this signaling pathway.

## Results

### Effect of BA on cell cycle arrest in HPS-stimulated piglet monocytes

**Fig. 1:**
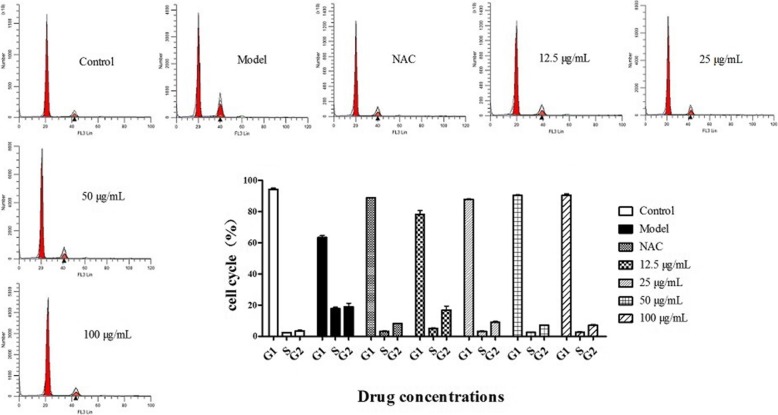
Effect of BA on cell cycle arrest in HPS-stimulated piglet monocytes. Cells (1.0 × 10^6^ cells per well) were treated with 0.5 mM NAC and BA (12.5, 25, 50 or 100 μg/ml) for 2 h and, after 6 h of incubation with HPS (final concentration 1.0 × 10^6^ CFU/ml), were stained with PI and analyzed using flow cytometry. The percentages of cells in different phases of the cell cycle were further analyzed by Modfit 4.1 software. All data represent means ± SD of three independent experiments

To determine the growth-inhibitory effect of HPS mediated by cell cycle arrest and the inhibitory effect of BA, the cell cycle distribution was determined using flow cytometry. Compared with the control group, the percentages of monocytes at G1-G0 phase decreased (*p* < 0.01), and at S and G2 phases increased (*p* < 0.01) after HPS stimulation (Fig. 1). The NAC and BA (12.5, 25, 50 or 100 μg/ml) treatment for 2 h significantly increased the proportion of cells at G1-G0 phase (*p* < 0.01) and suppressed the proportions at the S and G2 phases (*p* < 0.01).

The results revealed that HPS could inhibit the cell growth at G1-G0 phase dramatically (*p* < 0.01) in the model group. The inhibition could be declined obviously (*p* < 0.01) after 2 h of co-culture with 0.5 mM NAC or BA (12.5, 25, 50 and 100 μg/ml).

### Effect of BA on ROS generation in HPS-stimulated piglet monocytes

**Fig. 2:**
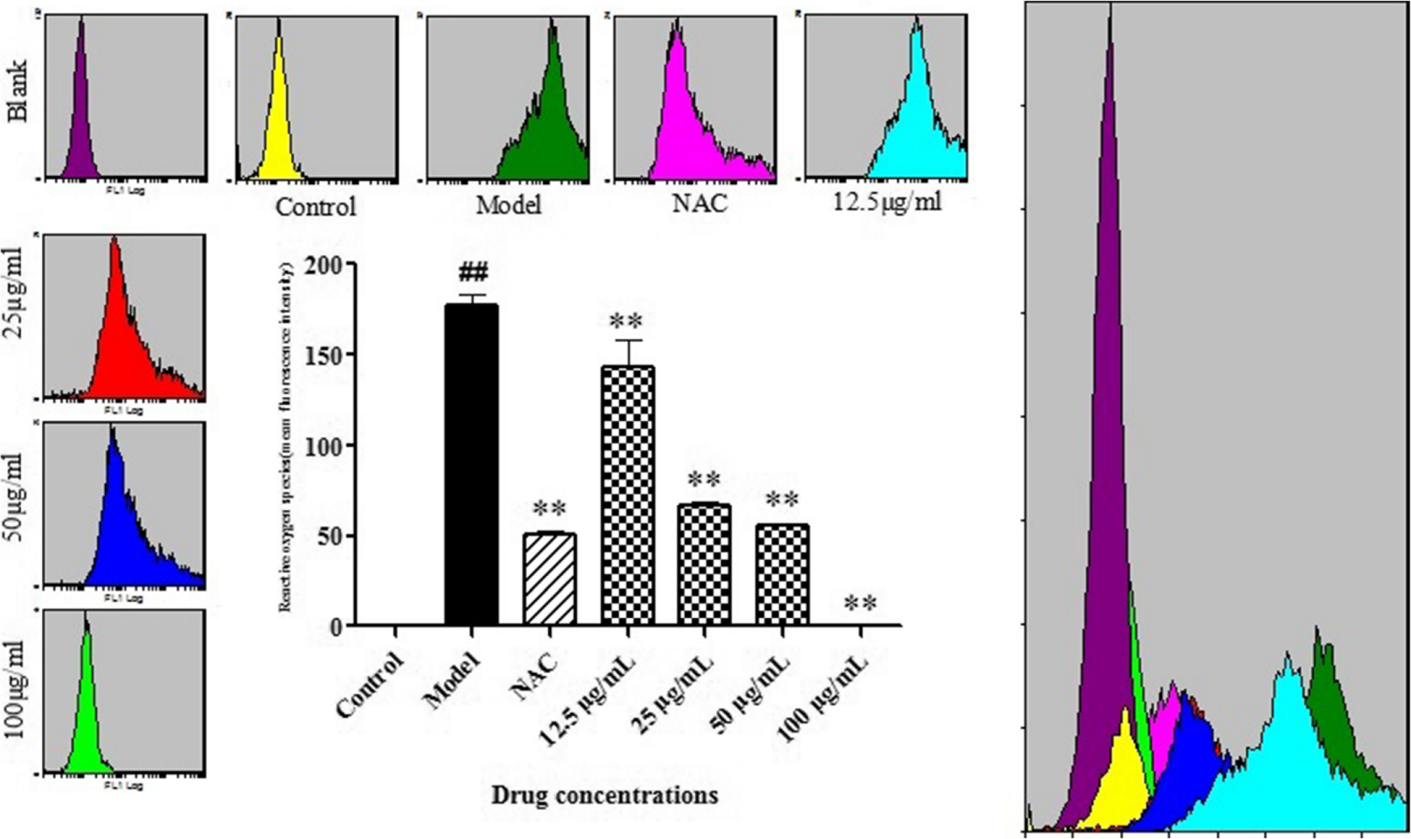
Effect of BA on ROS generation in HPS-stimulated piglet monocytes. Cells (0.8 ml, 1.0 × 10^6^ CFU/ml) were treated with 0.5 mM NAC and BA (12.5, 25, 50 or 100 μg/ml) for 2 h and, after 6 h of incubation with HPS (final concentration 1.0 × 10^6^ CFU/ml), were stained by DCFH-DA. The ROS levels in PMNP were determined and analyzed using flow cytometry. All data represent means ± SD of three independent experiments. ^##^ *p* < 0.01, compared with control group, ^**^ *p* < 0.01, related to HPS alone

The fluorescent probe DCFH-DA was used to detect intracellular ROS levels by flow cytometry in this study. Treatment with HPS caused a significant increase in ROS levels compared with control cells (*p* < 0.01; Fig. 2). To identify the role of ROS in mediating HPS-induced apoptosis, we used the ROS scavenger NAC – commonly used to inhibit ROS production. Pretreatment with NAC reduced HPS-induced ROS according to flow cytometry.

These results suggest that HPS-induced cell apoptosis in PMNP cells is potentially mediated by increased ROS accumulation. Interestingly, all concentrations of BA-treated groups had lower levels of ROS than the model group (*p* < 0.01; Fig. 2).

### Effect of BA on apoptosis in HPS-stimulated piglet’s monocytes

**Fig. 3:**
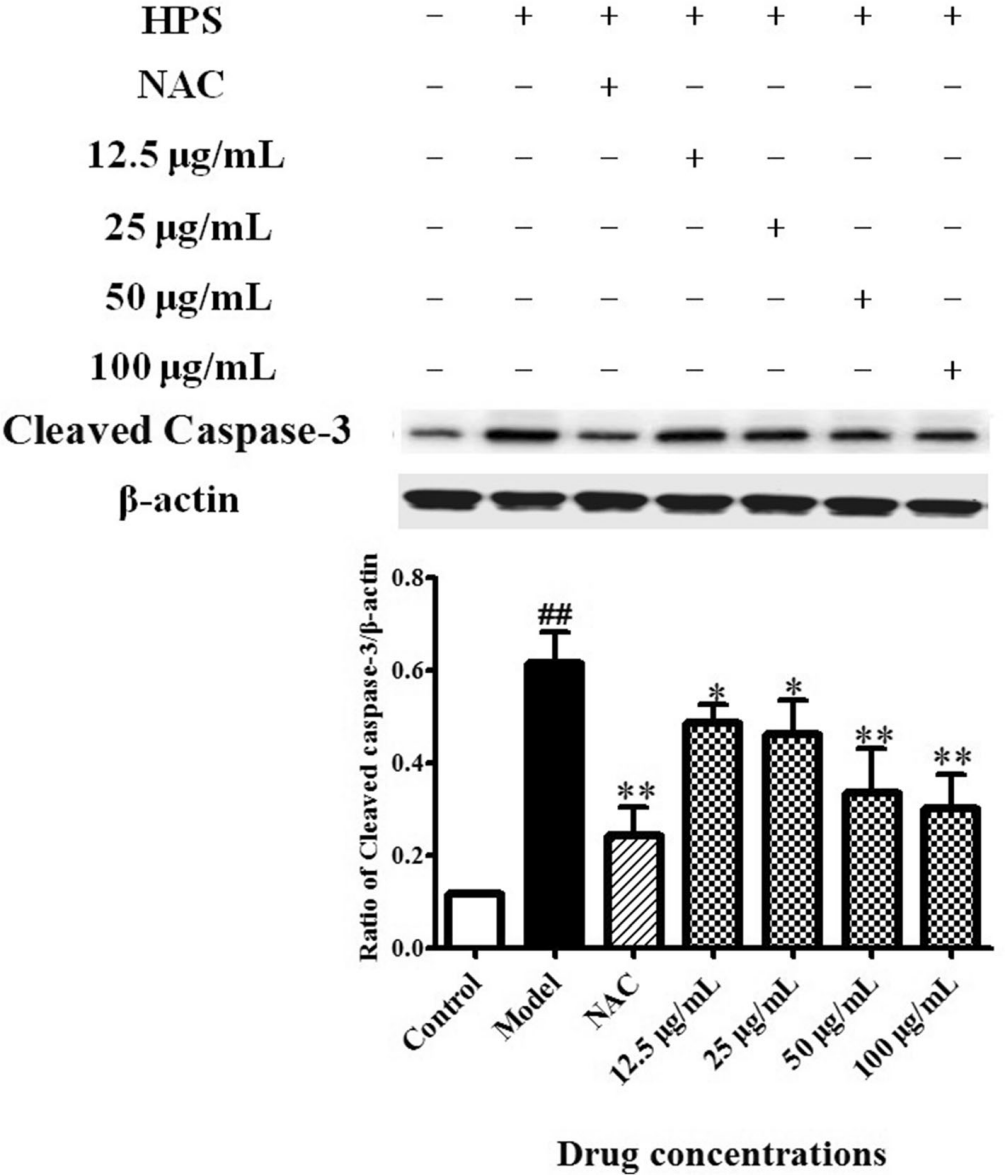
Effect of BA on apoptosis in HPS-stimulated piglet monocytes. The expression levels of cleaved caspase-3 were detected by western blotting. All data represent means ± SD of three independent experiments. ^##^ *p* < 0.01, compared with control group, ^*^ *p* < 0.05 and ^**^ *p* < 0.01, related to HPS alone

Because caspases work as executors in cell apoptosis, we examined the expression of caspase-related proteins. Western blot assay showed that HPS stimulation significantly enhanced the levels of cleaved caspase-3 in cells compared with the control (*p* < 0.01). Treatment with 0.5 mM NAC and BA of different concentrations (12.5 and 25 μg/ml, *p* < 0.05; 50 and 100 μg/ml, *p* < 0.01) down-regulated the cleaved caspase-3 protein expression in cells (Fig. 3). These data indicate that BA efficiently hindered apoptosis induced by HPS.

### Effects of BA on PKC–MAPK signaling pathways in HPS-stimulated piglet monocytes

**Fig. 4:**
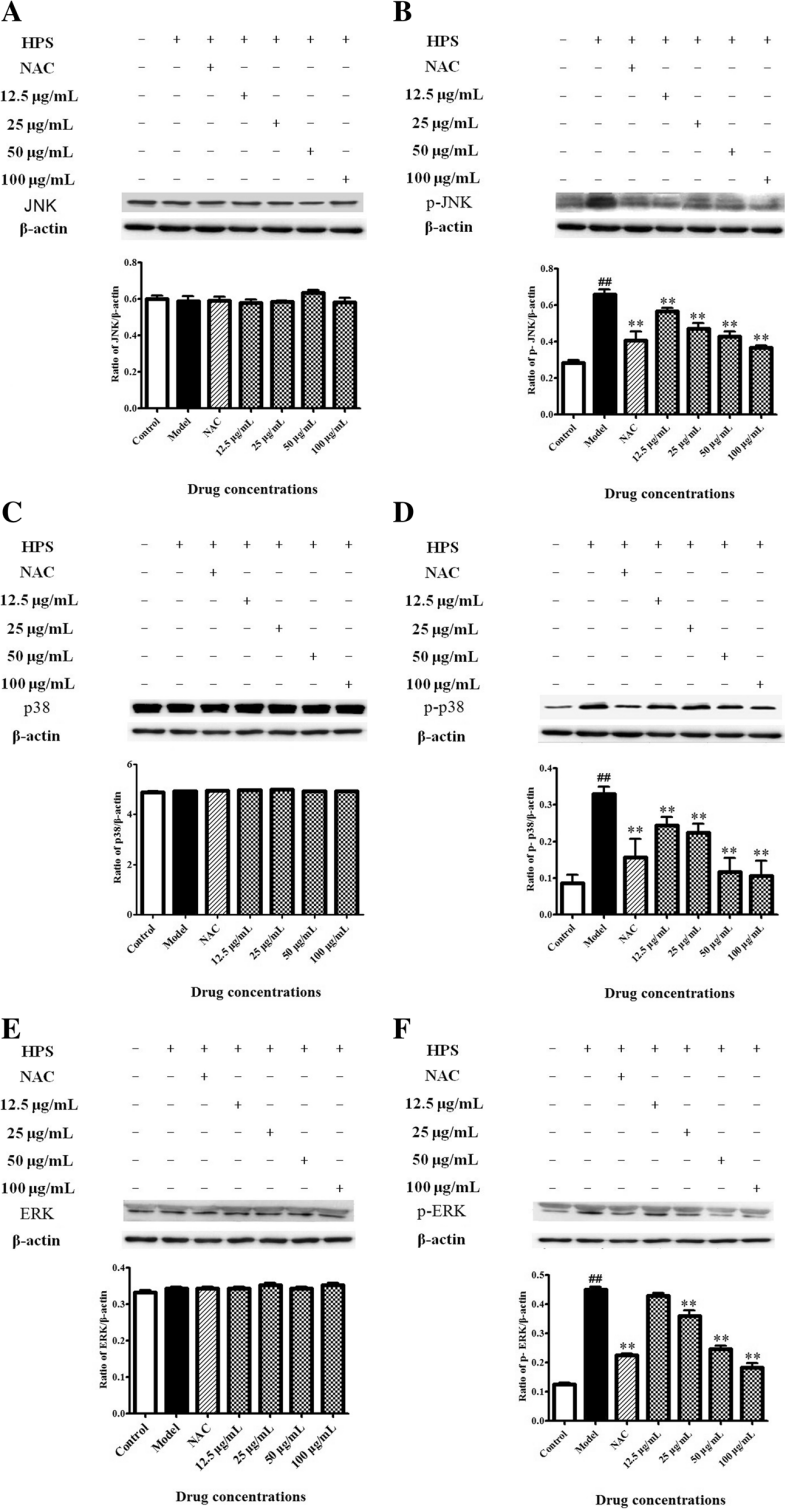
Effects of BA by activating MAPK in HPS-stimulated piglet monocytes. Cells were treated with 0.5 mM NAC and BA (12.5, 25, 50 or 100 μg/ml) for 2 h and 6 h of incubation with HPS (3.0 × 10^7^ CFU/ml). Western blot analysis of (**a**) JNK, (**b**) p-JNK, (**c**) p38, (**d**) p-p38 (**e**) ERK and (**f**) p-ERK. All data represent means ± SD of three independent experiments. ^##^ *p* < 0.01, compared with control group, ^**^ *p* < 0.01, related to HPS alone

Western blot analysis was performed to confirm whether HPS was involved in MAPK activation. Exposure to HPS for 6 h markedly elevated (*p* < 0.01) the phosphorylation levels of p38/JNK/ERK (Fig. 4b, d and f), yet the protein levels of JNK, p38 and ERK remained almost unified between the control and HPS groups (Fig. 4a, c and e). After monocytes were pretreated with NAC and BA, expressions of p-JNK and p-p38 proteins were down-regulated at all BA concentrations and 0.5 mM NAC; and the p-ERK protein levels decreased at BA concentrations of 25, 50 and 100 μg/ml and 0.5 mM NAC.

**Fig. 5:**
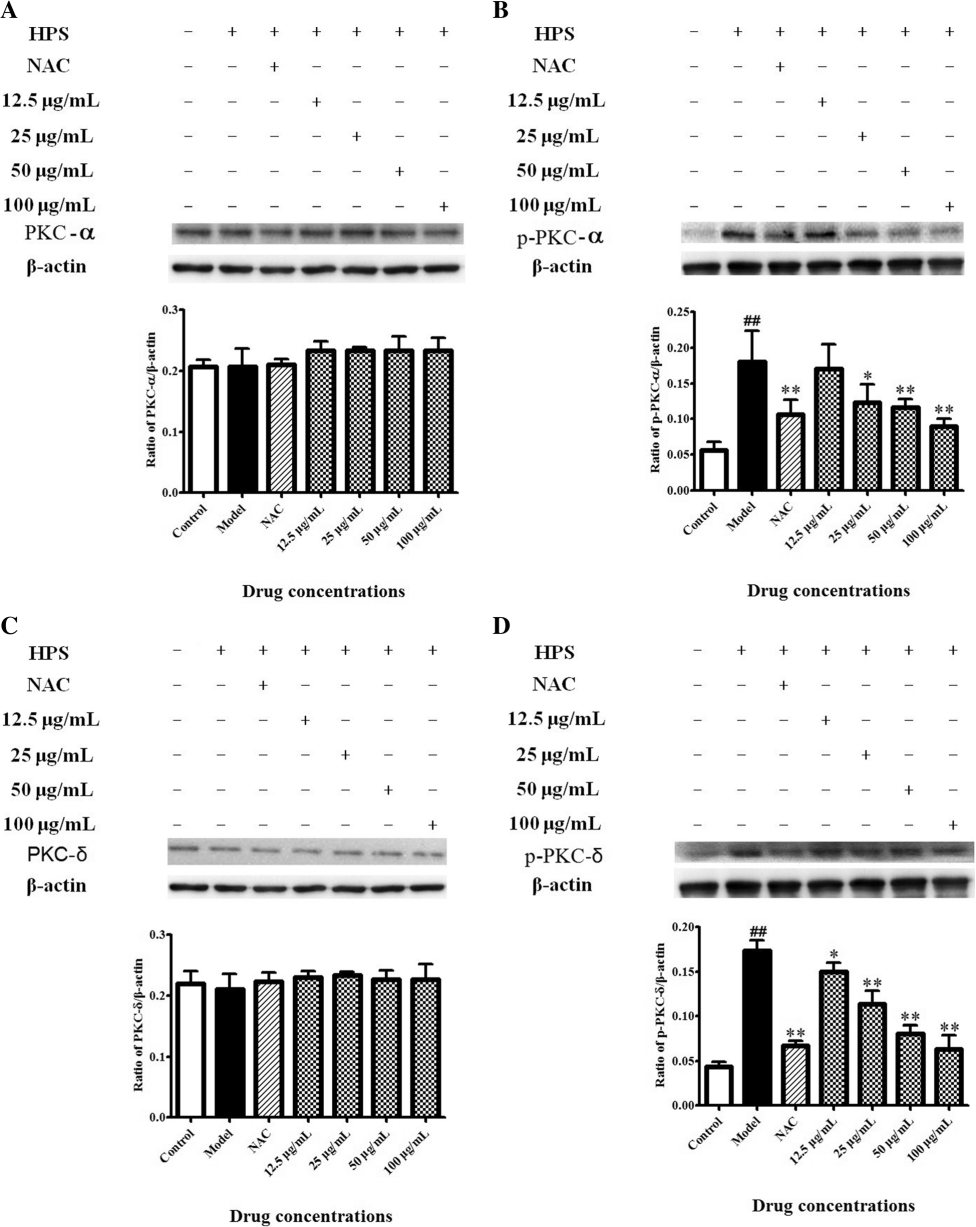
Effects of BA by activating PKC-mediation of HPS-stimulated piglet monocytes. Cells were treated with 0.5 mM NAC and BA (12.5, 25, 50 or 100 μg/ml) for 2 h and 6 h of incubation with HPS (3.0 × 10^7^ CFU/ml). Western blot analysis of (**a**) PKC-α, (**b**) p-PKC-α, (**c**) PKC-δ and (**d**) p-PKC-δ. All data represent means ± SD of three independent experiments. ^##^ *p* < 0.01, compared with control group, ^*^ *p* < 0.05 and ^**^ *p* < 0.01, related to HPS alone

Two isoforms of PKC were tested to find whether PKC could act directly in MAPK activation after HPS induction, and to determine the mechanism of action of BA. The HPS treatment significantly increased the phosphorylation levels of PKC-α and PKC-δ (*p* < 0.01), and the protein levels of PKC-α and PKC-δ did not significantly change. However, NAC and BA altered the effect of HPS in a dosage-dependent manner (Fig. 5).

### Effect of BA on activation of AP-1 family members in HPS-stimulated piglet monocytes

**Fig. 6:**
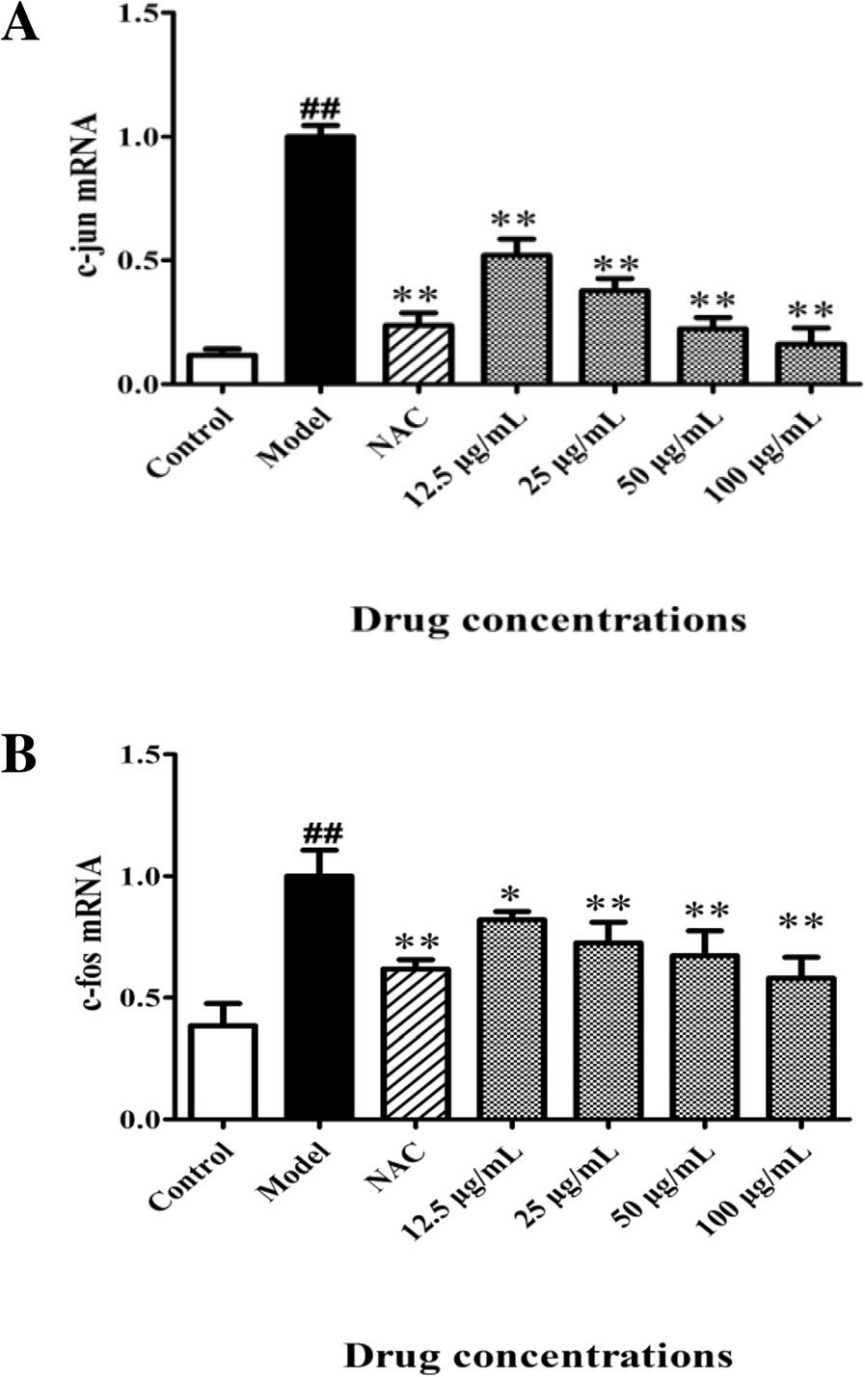
Effect of BA on activation of AP-1 family members in HPS-stimulated piglet monocytes. Cells were treated with 0.5 mM NAC and BA (12.5, 25, 50 or 100 μg/ml) for 2 h and 3 h of incubation with HPS (1.0 × 10^7^ CFU/ml). Q-RT-PCR analysis of (**a**) c-jun and (**b**) c-fos. All data represent means ± SD of three independent experiments. ^##^ *p* < 0.01, compared with control group, ^*^ *p* < 0.05 and ^**^ *p* < 0.01, related to HPS alone

Whether HPS stimulated AP-1 which is composed by c-Fos and c-Jun dimers was determined. RT-PCR revealed that the mRNA expressions of c-Jun and c-Fos significantly increased (*p* < 0.01) after HPS induction (Fig. 6); treatment with 0.5 mM NAC and BA at 25, 50 and 100 μg/ml both significantly (*p* < 0.01) reduced the expression of c-Jun and c-Fos.

## Discussion

In our earlier study, we demonstrated that BA could inhibit the activation of the NF-κB and NLRP3 inflammasome signaling pathway induced by HPS, and observed the protective effect of BA on ROS generation and cell apoptosis, using fluorescence microscopy [4]. That was in complete agreement with the results by flow cytometry in this study. In the present article, we extended our observations that mechanisms underlying these protective effects of BA appeared to deactivate the PKC–MAPK signal pathway, which may be associated with the production of ROS, the cell cycle progression and cellular apoptosis in PMNP after HPS stimulation. Our results showed that PMNP infected with HPS exhibited an increase in ROS and transcriptional expression of cleaved caspase-3, thus inducing apoptosis. Moreover, BA could inhibit the activation of the PKC–MAPK signaling pathway triggered by HPS in PMNP.

Excessive ROS accumulation can also lead to oxidative DNA damage, resulting in cell cycle process arrest or delay [20]. Our data demonstrated that HPS infection induced obvious accumulation of cells in S and G2 phases, and dramatically decreased those in the G1-G0 phase, indicating activation of apoptosis. Furthermore, we also found that BA and NAC significantly decreased cells in the G1-G0 phase and relieved the cell cycle arrest in S and G2 phases. These results revealed that action in relieving cell cycle arrest may be one mechanism of the anti-apoptosis effect of BA.

ROS have been demonstrated to be an early signal mediating apoptosis via activating mitochondrial pathways [21]. HPS as a Gram-negative bacterium has an outer membrane containing LPS, and LPS-mediated mitochondrial apoptosis of monocytes has been previously observed [22]. HPS and LPS could both induce ROS generation in our previous experiments [4, 11]. Liu et al. reported that ROS could activate MAPK pathways [23]. In order to identify the role of ROS in mediating HPS-induced apoptosis in PMNP, we used NAC (an ROS scavenger) as a positive control to evaluate the effect of BA on inhibiting ROS production and the activation of MAPK induced by HPS [24]. As expected, ROS production was significantly enhanced by HPS treatment, and the pretreatment of cells with 0.5 mM NAC for 2 h significantly inhibited HPS-induced ROS production. Flow cytometry showed that co-treatment with NAC rescued HPS-induced apoptosis. These results suggest that HPS-induced cell apoptosis in PMNP is potentially mediated by increased ROS accumulation. In addition, pretreatment with NAC resulted in lower levels of p-p38, p-ERK and p-JNK in HPS-stimulated cells. The expression of cleaved caspase-3 also decreased in HPS-stimulated cells in the presence of NAC. Taken together, inhibition of ROS suppressed the HPS-induced activation of MAPK and the following mitochondrial signal pathways, suggesting that ROS is upstream of the MAPK-mediated mitochondrial signal pathway in HPS-induced apoptosis in PMNP. Interestingly, we found that BA could effectively reduce intracellular excessive ROS generation in PMNP, and so inhibit apoptosis. The data in this study showed that BA at mid-dosage had a similar effect to NAC in inhibiting ROS production, and was more effective at high-dosage. So, we speculate that one protective mechanism of BA in relation to apoptosis is the down-regulation of HPS-induced ROS; due to its anti-oxidant properties, BA plays a protected role in mediating apoptosis induced by oxidative stress. The action of BA in PMNP could be similar to that of NAC.

Our previous studies demonstrated that LPS infection could activate the MAPK signaling pathway, and induce IL-6 and TNF-α overproduction [22]. MAPK including ERK, JNK and p38 form a signal cascade to link several cell functions such as proliferation, differentiation and apoptosis [25]. HPS-induced changes in p38 MAPK phosphorylation were reversed by NAC and BA pretreatment, suggesting that BA potentially mediates activation of the p-p38 MAPK pathway induced by HPS. Taken together, these results suggest that BA inhibited the activation of p-p38MAPK in its contribution to apoptosis. JNK plays an important role in regulating cell viability, and targeted disruption of JNK results in defects in apoptosis [26]. Several studies have demonstrated that p38 MAPK and JNK are activated by ROS or pro-inflammatory cytokines and promote apoptosis [27–29]. One essential member of the MAPK family of signaling cascades, the ERK pathway, is associated with cellular inflammation, oxidative stress and apoptosis [30]. In our study, BA treatment significantly reduced HPS-induced p-ERK expression, with a similar influence on JNK phosphorylation.

PKC acts as a crucial ROS-sensitive kinase and activates MAPK signaling under a range of different stimulations [13]. Treatment with the PKC inhibitor GF-109203X blocks TPA-induced ERK and JNK protein phosphorylation, indicating that activation of PKC was followed by activation of MAPK [25]. PKC is a serine/threonine family of 10 related isoforms (PKCs) and is divided into three subclasses – conventional (cPKC-α, −β and -γ), novel (nPKC-δ, −ε and -θ) and atypical (aPKC-ζ and -λ) – which are produced by different genes, and play an important role in immune signaling [31, 32]. PKC-α plays an important role in the apoptosis of some tumor cells. PKC-δ can mediate JNK activation in response to DNA damage [33]. MAPKs including ERK2, p38 MAPKs and JNKs are activated in response to IFNα in some cells via PKC-δ [34]. In the present study, we investigated only two isoforms of PKC, and future work should include more isoforms involved in HPS-induced apoptosis of PMNP. One important finding in this study was activation of phosphorylation levels of PKC-α and PKC-δ in PMNP subjected to HPS, and pretreatment with BA significantly decreased activation of both proteins. This suggests that the protective effect of BA against HPS-induced apoptosis occurs through activation of the PKC–MAPK pathway. Another important and intriguing question would be how HPS activates PKC. Some reports found that both TLR2 and TLR4 mediate phosphorylation of PKC-α [35]. PKC-α could regulate NF-κB and MAPK activation downstream of TLR2 in murine DC [36]. Distinct intracellular signaling pathways leading to MAPK activation are induced by TLR4 and TLR2 stimulation and regulated by PKC-α/β [37]. HPS infection activated TLR1, 2, 4 and 6 mediating the p38/JNK MAPK signaling pathways linked to inflammation and CCL4 transcription in PK-15 cells [12]. Extracellular signals are transduced into macrophages via TLR4 with LPS treatment to induce innate immunity in macrophages, then activating the MAPK pathway by phosphoinositide 3-kinase, PKC and protein-tyrosine kinases [38]. Our results indicate that PKC is a key signaling node participating in a HPS-mediated apoptosis mechanism in PMNP, and might act as a crucial link between the TLR and subsequent MAPK signaling activations. The detailed mechanism between the activation of TLRs and PKC–MAPK requires further investigation.

The AP-1 transcription factors are involved in both the induction and prevention of apoptosis, and the first indication was observations linking the induction of c-Fos and c-Jun [39]. Activated PKCs and MAPKs may also participate in transcriptional regulation of AP-1 [14, 15]. MAPK families serve an important role in activation of AP-1. ERK enhances AP-1 activation through c-Fos, whereas JNK leads to the phosphorylation of c-Jun [40]. In our study, HPS induced the expression of c-Jun and c-Fos, which were attenuated by NAC and BA treatment.

One important outcome of our study is the illustration of the mechanism of apoptosis caused by HPS infection, through ROS–mitochondrial and TLR–PKC signaling pathways in PMNP for the first time. We also demonstrated apoptosis via the regulation of caspase-3 protein and activation of the AP-1 transcription factor.

## Conclusion

This study brings new insights into the molecular pathogenesis of HPS-induced apoptosis. The anti-apoptosis effect of BA was associated with its ability to regulate the PKC–MAPK signaling pathways. Combined with our previous findings that BA has anti-inflammatory activity through inhibiting the activation of NF-κB and NLRP3 inflammasome signaling pathway in PMNP treated with HPS, we provide evidence that BA could be a novel therapeutic intervention for the treatment of Glasser’s disease.

## Methods

### Bacterial strain, growth conditions and drugs

The HPS SH0165 strain, a highly virulent strain of serovar 5, was isolated and grown refer to our previous report [4].

BA was obtained from the National Institute for Food and Drug Control (Beijing, China, B110715–201318), dissolved and diluted using RPMI-1640 medium (Gibco, NY, USA) without any other solvent.

*N*-Acetylcysteine (NAC) was purchased from Sigma Aldrich (Lot# SLBK6227V), dissolved in RPMI-1640 at a final concentration of 0.5 mM and stored at − 20 °C.

### Isolation and culture of peripheral blood monocytes

Three 30-day-old crossbred healthy piglets (Duroc × Landrace × Large White) which were negative for detection of antibody against HPS by INGEZIM Haemophilus 11. *H. parasuis*. K1 (Ingezim, Spain) obtained from Hubei Tianzhong Animal Husbandry Co. Ltd. (Wuhan, China), weighing 9–10 kg, were used for in vitro experiments. The protocol for animal use for this research was approved by the China Hubei Province Science and Technology Department (permit number SYXK (ER) 2010–0029). All experimental animals were given pentobarbital sodium (80 mg/kg·bw) by intramuscular administration at the end of the experiments for euthanizing. After 10–15 min, all the animals were anaesthetised and unconscious.

The blood used for monocytes was isolated and cultured for the following studies using a method previously described [11]. The blood monocyte infection model of HPS was constructed in our previous research [4].

### Cell cycle distribution assay

Cell cycle distribution was determined using flow cytometry. Cells were plated in 60-mm single-well culture plates (1.0 × 10^6^ cells per well) and pretreated with 0.5 mM NAC, various concentrations of BA (12.5, 25, 50 or 100 μg/ml) or an equal volume of RPMI-1640 for 2 h. Cells were harvested after 6 h of incubation with HPS (final concentration 1.0 × 10^6^ CFU/ml) and then fixed in ice-chilled 70% ethanol overnight at 4 °C. After centrifugation, the suspension was removed. Cells were stained with PI/RNase staining buffer for 15 min at 37 °C with 5% CO_2_ according to the manufacturer’s instructions (Becton-Dickinson Biosciences), and analyzed by flow cytometry. The percentages of cells in different phases of the cell cycle were further analyzed by Modfit 4.1 software.

### Detection of intracellular ROS levels

The piglet monocytes were inoculated in 24-well plates (0.8 × 10^6^). RPMI-1640 medium containing BA (12.5, 25, 50 and 100 μg/ml) was added and cells incubated for 2 h followed by 6 h of HPS stimulation (final concentration 1.0 × 10^6^ CFU/ml). The monocytes in the NAC group were treated with 0.5 mM NAC, the model group was in medium containing HPS without drugs, and the blank control group was treated with an equal volume of medium without HPS or any drugs. The 10 μM DCFH-DA and 5 μM DHE were added for 30 min at 37 °C with 5% CO_2_ according to the ROS manufacturer’s instructions (Nanjing Jiancheng Bioengineering Institute, E004). The levels of intracellular ROS were confirmed using a flow cytometer equipped with FC 500 image software to detect fluorescence intensity of cells (Cytomics Fc500 MCL, Beckman Coulter, USA). All experiments were repeated independently three times.

### Western blot analysis

Cells (3.0 × 10^7^) were treated with 0.5 mM NAC, BA (12.5, 25, 50 or 100 μg/ml) and an equal volume of RPMI-1640 medium for 2 h. HPS (3.0 × 10^7^ CFU/ml) was added and the protein was extracted after 6 h using RIPA Lysis Buffer Strong (P0013, Beyotime Biotechnology, Shanghai, China). The protein concentration was determined by BCA protein assay kit (P0012, Beyotime Biotechnology). The proteins were separated by 10% SDS-PAGE and transferred onto a PVDF membrane (60 mA, 1 h). The PVDF membrane was blocked with 5% skimmed milk at 20 °C for 3 h, then incubated with the primary antibody (containing 5% BSA TBS-T solution, 1:1000 dilution, Anti *β*-actin, cleaved caspase-3, JNK, p-JNK, p38, p-p38, ERK, p-ERK, PKC-α and p-PKC-δ, Cell Signaling Technology, MA, USA; and p-PKC-α and PKC-δ, Sigma, USA) overnight at 4 °C and the secondary antibody (containing 5% skimmed milk TBS-T solution, 1:2000 dilution; Cell Signaling Technology) at room temperature for 3 h. Afterwards, the membrane was visualized by ECL solution (Thermo Pierce ECL, USA) using a ChemiDoc MP Imaging System (Bio-Rad, Hercules, CA, USA). The *β*-actin was used as an inner loading control. Gray value was analyzed and the relative expression level of protein was obtained.

### Total RNA extraction and Q-RT-PCR

Total RNA was extracted with Trizol reagent after the monocytes (1.0 × 10^7^) were treated with 0.5 mM NAC, BA (12.5, 25, 50 or 100 μg/ml) and an equal volume of RPMI-1640 medium for 2 h followed by 3 h of HPS stimulation (1.0 × 10^7^ CFU/ml).

The RNA was reverse-transcribed into cDNA using the reverse-transcription kit following the manufacturer’s instructions (Takara Biotechnology, Kusatsu, Japan), and quantified by SYBR Premix Ex Taq kit (Takara Biotechnology). Specific expression primers for c-Jun, c-Fos and *β*-actin as a reference gene were designed using Primer 6.0 software. Primer sequences and conditions of RT-PCR are shown in Table 1. The quantitative results for fluorescence were calculated by 2^–ΔΔCt^ using the normalization method.

**Table 1:** Sequence of primers used in current investigation by Q-RT PCR

Gene	Primer	Sequence 5′ → 3’	Tm (°C)	Product Size (bp)
c-Jun	Forward	AGAATCCGAAGGGAAAGGA	53.4	20
	Revrese	CTTCTCCTTCAGCAGGTTGG	57.4	20
c-Fos	Forward	GCTGACAGATACACTCCAAGCGG	61.3	23
	Revrese	AGGAAGACGTGTAAGTAGTGCAG	57.8	23

### Statistical analysis

Results are expressed as mean ± SD. Data were analyzed using ANOVA followed by LSD method for independent means using SPSS Statistics 17.0 (IBM, Armonk, NY, USA). A value of *p* < 0.05 was considered significant.

## Abbreviations

AP-1: Activator protein-1
BA: Baicalin
ERK: Extracellular signal-regulated kinase
HPS: *Haemophilus parasuis*
JNK: c-Jun amino-terminal kinase
LPS: Lipopolysaccharide
MAPK: Mitogen-activated protein kinase
NAC: *N*-Acetylcysteine
NF-κB: Nuclear factor-kappa B
PAM: Porcine alveolar macrophage
PK-15: Kidney epithelial
PKC: Protein kinase C
PMNP: Piglet mononuclear phagocytes
ROS: Reactive oxygen species
TLRs: Toll-like receptors
